# Development a hydrolysis probe-based quantitative PCR assay for the specific detection and quantification of *Candida auris*

**DOI:** 10.18502/cmm.6.3.4665

**Published:** 2020-09

**Authors:** Hadis Jafarian, Hossein Khodadadi, Parisa Badiee

**Affiliations:** 1 Department of Medical Parasitology and Mycology, School of Medicine, Shiraz University of Medical Sciences, Shiraz, Iran; 2 Alborzi Clinical Microbiology Research Center, Shiraz University of Medical Sciences, Shiraz, Iran

**Keywords:** *Candida auris*, Quantification, Real-time PCR Introduction

## Abstract

**Background and Purpose:**

*Candida auris* is an emerging multidrug-resistant pathogen. The identification of this species with the conventional phenotypic or biochemical mycological methods may lead to misidentification. Molecular-based species-specific identification methods such as quantitative real-time polymerase chain reaction (qPCR) facilitate a more reliable identification of *C. auris* than mycological methods. Regarding this, the present study aimed to develop a hydrolysis probe-based qPCR assay for the rapid, accurate identification of *C. auris*.

**Materials and Methods:**

The internal transcribed spacer 2 regions in the nuclear ribosomal DNA of *C. auris* and other related yeasts were assayed to find a specific PCR target for *C. auris*. A 123-base-pair target was selected, and primers and a probe were designed for hydrolysis probe-based real-time PCR with TaqMan chemistry. Ten-fold serial dilutions of *C. auris* ranging from 106 to 100 CFU/mL were prepared to establish a standard curve to quantify the yeast.

**Results:**

The qPCR assay was able to identify and quantify *C. auris* with a detection limit of 1 *C. auris* CFU per reaction. Specificity was confirmed by the non-amplification of the sequences belonging to other* Candida* species, yeasts, molds, bacteria, or human DNAs. The standard curve of the assay showed a highly significant linearity between threshold values and dilution rates (R^2^=0.99; slope=−3.42).

**Conclusion:**

The applied qPCR assay facilitated the rapid and accurate identification and quantification of emerging opportunistic *C. auris*. Therefore, considering the promising test validation results, we succeeded to develop a rapid and accurate hydrolysis probe- based qPCR assay for the screening and identification of *C. auris*.

## Introduction

*Candida auris* is an emerging multidrug- resistant pathogen. This yeast, isolated and identified from the ear discharge of a Japanese patient in 2009 [ 1 ], is an ascomycetous species belonging to the order Saccharomycetales and closely related to *C. haemulonii* and *C. pseudohaemulonii* [ 1 ]. The species has spread rapidly around the world and has been isolated in Japan [ 1 ], South Korea [ 2 ], India [ 3 ], Pakistan [ 4 ], Kuwait [ 5 ], Oman [ 6 ], South Africa [ 7 ], Colombia [ 8 ], Venezuela [ 4 ], the UK [ 9 ], Canada [ 10 ], some European countries [ 11 ], and recently Iran [ 12 ]. It has been identified as a causative agent for hospital- acquired infections and is resistant to most available antifungal drugs. The yeast can remain on environmental surfaces in hospitals for prolonged periods and may be passed on to other patients [ 13 ].

The identification of *C. auris* with routine mycological methods (e.g., germ tube and chlamydospore production or sugar assimilation and fermentation) is time-consuming and may lead to misidentification [ 3 , 4 , 14 , 5 ]. These methods often misidentify *C. auris* isolates as a range of other *Candida* species, including *C. haemulonii*, *C. famata*, *C. sake*, *C. catenulate*, *C. lusitaniae*, *C. guilliermondii*, and *C. parapsilosis*, or as other yeasts, such as *Rhodotorula glutinis, Rhodotorula mucilaginosa,* and *Saccharomyces species* [ 4 , 5 , 7 , 15 - 19 ]. Because *C. auris* shows a close phylogenetic relation to the *C. haemulonii* complex, some *C. auris* isolates may have been misidentified as *C. haemulonii* in previous reports [ 20 ].

Sequencing the D1/D2 domain in large ribosomal subunits and internal transcribed spacer 1 and 2 (ITS1,ITS2) regions of rRNA genes has proven helpful in the identification of yeast species. This method is also a major technique for the identification of fungi in research laboratories. However, it has not been established as a routine method in clinical laboratories for the identification of *Candida* species, including *C. auris* [ 7 , 21 , 2 ]. Matrix-assisted laser desorption ionization time of flight mass spectrometry (MALDI- TOF MS) is a broad-spectrum tool facilitating the identification of all yeasts rapidly and accurately [ 23 , 24 ]. However, it is associated with some limitations. The method can identify only organisms included in its reference database, and there is a potential risk for the misidentification of some uncommon yeast, such as *C. auris* [ 25 ]. Another important limitation is the expense and unavailability of the instrument in resource-limited regions [ 15 ].

When MALDI-TOF MS capability is unavailable, developing specific molecular identification methods based on DNA amplification is a potential aid to identifying *C. auris*. Recently, several in-house PCR- based methods, including real-time PCR assays with hydrolysis probes or melting curve analysis, have been introduced to detect this species [ 26 - 28 ]. The development of a specific quantitative PCR assay for *C. auris* can play an important role in the rapid and accurate identification of this species and is thus potentially useful in time-limited situations and diagnostic laboratories without access to advanced systems, such as MALDI-TOF MS. The sequential quantification of infection load caused by the yeast may help improve treatment monitoring in patients.

The present study involved the development and validation of a hydrolysis probe-based (TaqMan Probe) quantitative real-time PCR (qPCR) assay compatible with the Minimum Information for Publication of Quantitative Real-Time PCR Experiments guidelines for the rapid and accurate screening and identification of *C. auris*.

## Materials and Methods

**Primer and probe design**

The ITS sequences of 12 *C. auris* strains (belonging to different clades and isolated from different parts of the word), and the ITS sequences of species closely related to *C. auris*, and other common yeasts deposited in GenBank were downloaded. The sequences were multiple-aligned and investigated to find a specific region for use in designing a hydrolysis probe-based qPCR assay for *C. auris*. Multiple alignments were performed using Geneious software (Biomatters, Auckland, New Zealand).

A set of primers and a hydrolysis probe (TaqMan Probe) were designed for the selected highly specific ITS region of *C. auris* ) GenBank accession number AB375772.1), using GenScript real-time PCR (TaqMan) primer design (www.genscript.com/tools/real-time-pcr-tagman-primer-design-tool). The expected length of the amplicon from the nucleotide sequence of *C. auris* was 123 bp. The forward and reverse primers used in this study were Cau-qRT-F: 5′- AGCGTGATGTCTTCTCACCA-3′ and Cau-qRT-R: 5′-GCGGGTAGTCCTACCTGATT-3′, and the probe oligonucleotide sequence was Cau-qRT-P: 5′- TGAATGCAACGCCACCGCGA-3′.

The specificity of the designed primers was checked *in silico* using the basic local alignment search tool (BLAST) against the National Center for Biotechnology Information (NCBI) database to verify that they did not amplify the sequences of species other than *C. auris*. Eurofins Genomics (Ebersberg, Germany) synthesized the primers and probe oligonucleotides. The Cau-qRT-P hydrolysis probe was double-labeled with the 5'- reporter dye 6-carboxyfluorescein and the downstream 3'-quencher dye carboxytetramethylrhodamine.

**Fungal strains**

Different fungal and bacterial strains (Table 1) were used to check the sensitivity and specificity of the amplification method. Three standard strains of *C. auris* (i.e., CBS 14916, CBS 10913, and CBS 12372, isolated from patients in different geographic regions of the world) and one *C. haemulonii* strain (CBS 7801) were obtained from the CBS-KNAW culture collection (Westerdijk Fungal Biodiversity Institute, Utrecht, Netherlands). Other standard fungal or bacterial strains and human genomic DNAs were included in the specificity evaluation (Table 1). In addition, a total of 104 archived clinical yeast isolates, including 49 *C. albicans*, 15 *C. parapsilosis*, 10 *C. famata*, 5 *C. krusei,* 4 *C. glabrata,* 4 *C. tropicalis,* 2 *C. lusitaniae*, and 15 unknown species, were tested.

**Table 1:** Fungal and bacterial strains used to determine specificity of primers and probe

Species	Strains
*Candida albicans*	ATCC 10231
*C. glabrata*	ATCC 2001
*C. parapsilosis*	ATCC 22019
*C. auris*	CBS 12372, CBS 14916,
	CBS 10913, MK123931.2
*C. haemulonii*	CBS 7801
*Escherichia coli*	ATCC 25922
*Klebsiella pneumoniae*	ATCC 13883
*Staphylococcus aureus*	ATCC 25923
*Staphylococcus epidermidis*	ATCC 12228
*Saccharomyces cerevisiae*	PTCC 5177, ATCC 2601
*C. krusei*	Clinical isolate
*C. kefyr*	Clinical isolate
*Rhodotorula species*	Clinical isolate
*Aspergillus niger*	Clinical isolate
*Aspergillus fumigatus*	Clinical isolate
*Aspergillus flavus*	Clinical isolate
*Penicillium species*	Clinical isolate

The yeasts were isolated from patients suspected of having candidemia by BACTEC blood culture (BD, Franklin Lakes, New Jersey, USA) and identified according to biochemical tests with API 20C AUX (bioMérieux, Craponne, France) and their PCR- restriction fragment length polymorphism patterns. The yeasts were stocked in the mycology laboratory collection at Professor Alborzi Clinical Microbiology Research Center, Shiraz, Iran. Fungal strains were grown on Sabouraud dextrose agar (SDA), containing 50 mg/L chloramphenicol, and bacterial strains were cultured on Mueller-Hinton agar prior to DNA extraction.

**DNA extraction**

Total yeast DNA was extracted from fresh colonies as described previously [ 29 ]. Briefly, one yeast colony was picked from the plate, and cells were suspended in 100 μL double-distilled water and heated at 100°C for 10 min. The suspension was centrifuged for 3 min at 2000 g, and the supernatant was stored at -20°C until use as the DNA template in amplification reactions. The DNA of filamentous fungi was extracted with the phenol-chloroform method as described by Zarinfar et al. [ 30 ]. A 100-μL portion of a suspension of each bacterial strain with a turbidity of McFarland No. 1 was heated at 100°C for 10 min and used as the bacterial DNA template. The purities of DNA were assessed by evaluating the absorbance ratio at 260 and 280 nm (A260/A280) on a NanoDrop 2000/2000c spectrophotometer (Thermo Fisher Scientific, Waltham, Massachusetts, USA).

**Conventional polymerase chain reaction**

To check and optimize the annealing temperature for the designed primers, conventional PCR was performed with the same set of primers (i.e., Cau-qRT- F and Cau-qRT-R). The PCR mixtures contained 3 µL fungal DNA template and 1× Taq DNA polymerase Master Mix RED with 2.0 mmol/L MgCl2 (Ampliqon, Odense, Denmark) and 5 pmol of each primer. Thermal cycling was carried out in a T100 Bio-Rad PCR machine (Bio-Rad, California, California, USA). The thermal cycling steps consisted of initial denaturation at 94°C for 5 min, 30 cycles of denaturation at 94°C for 30 sec, annealing at a temperature gradient of 56-64°C for 45 sec, and extension at 72°C for 45 sec, followed by a final extension step at 72°C for 7 min. All PCR reactions were performed in a total volume of 25 µL in duplicate. *Candida auris* DNA as a positive control and double-distilled water as a negative control were processed in parallel with the test samples to identify possible false-positive or false-negative results, as well as contamination in each run of the assay. Five microliters of PCR products was electrophoresed on 1.5% agarose gels. The gels were stained with 0.5 µg/mL ethidium bromide and examined for bands with predicted sizes in a UV transilluminator gel documentation system (UVTECH, Cambridge, UK).

**Real-time polymerase chain reactions**

TaqMan real-time PCR was performed with the Cau-qRT-P probe and the Cau-qRT-F and Cau-qRT-R primers in a total volume of 20 µL. Each reaction consisted of 1× HOT FIREPol Probe Universal qPCR Mix Plus ROX (Solis BioDyne, Tartu, Estonia), 0.3 μmol/L of each primer, 0.15 μmol/L of probe, and 2 μL of DNA template. Two non-template controls (NTC), containing molecular-grade water instead of DNA, and positive controls, containing DNA extracted from *C. auris*, were included in each run, and experiments were performed in duplicate. Thermal cycling was carried out with the Applied Biosystems 7500 (Applied Biosystems Inc, Beverly, Massachusetts, USA) real- time PCR system. It consisted of a single initial denaturation step of 10 min at 95°C, followed by 45 cycles of 95°C for 5 sec and 63°C for 30 sec. The fluorescent signal collection point of the instrument software was adjusted for each extension step.

The potential usefulness of using primers in a SYBR Green-based real-time PCR assay was also investigated using the same Cau-qRT-F and Cau-qRT- R primers. The PCR assays with SYBR Green were carried out with PCR mixtures consisting of 1× SYBR Green Master Mix (Applied Biosystems), 5 µmol/L of each primer, and 2 µL of extracted DNA in a total volume of 20 µL. Two NTCs, containing molecular- grade water instead of DNA, and positive controls, containing DNA extracted from *C. auris*, were included in each run. Real-time PCR experiments were performed in duplicate for each strain or sample. Furthermore, the amplifications were performed on the Applied Biosystems 7500 real-time PCR instrument.

The PCR protocol included an initial denaturation step at 95°C for 10 min, followed by 40 cycles of denaturation at 95°C for 15 sec and an annealing/extension step at 60°C for 1 min. The data collection point was reset for each annealing/extension step. The cycle threshold and melting curve were determined automatically.

**Specificity of hydrolysis probe real-time polymerase chain reaction**

The specificity of the hydrolysis probe and real- time PCR primers was first checked in silico by subjecting the selected primers and probes to a BLAST test (http://blast.ncbi. nlm.nih.gov). Then, the experiments were performed with DNA templates from different *C. auris*, fungal, bacterial, and human DNAs (Table 1).

**Sensitivity of hydrolysis probe real-time polymerase chain reaction**

Fresh colonies of *C. auris* were obtained by growing a standard strain of the yeast for 48 h on SDA at 37°C. The harvested colonies were washed twice with sterile normal saline and collected by centrifugation. A suspension of the washed cells was prepared in normal saline, and cells were counted in a hemocytometer chamber. The yeast suspension count was adjusted to 10^7^ cells/mL. Ten-fold serial dilutions ranging from 10^6^ to 10^0^ CFU/mL were prepared to determine the analytical sensitivity of the assay [ 27 , 28 ]. To check the CFU count of viable *C. auris* in each dilution, 100 µL of each dilution was cultured on SDA plates. The DNA was extracted from 100 μL of each dilution and used to study the analytical sensitivity and limit of detection (LOD) of the assay. A standard curve was plotted according to the threshold values of serial dilutions and their CFU counts and was also used to determine PCR efficiency. The LOD of real-time PCR assays was defined as the lowest concentration at which a positive result was detected in more than 90% of the experiments. Each reaction was performed in triplicate.

## Results

**Primer and probe design**

Sequence homology analysis confirmed the specificity of the primers designed for *C. auris* and its related species *C. haemulonii*, while the designed probe showed specificity only for *C. auris*.

**DNA extraction**

Extracted DNAs indicated a change in A260/A280 ratio from 1.7 to 2.1, an acceptable quality for use in amplification reactions. The extracted DNA was used in amplification reactions if showing a concentration from 100 pg to 1 μg of genomic DNA.

**Conventional polymerase chain reaction**

**Figure 1:**
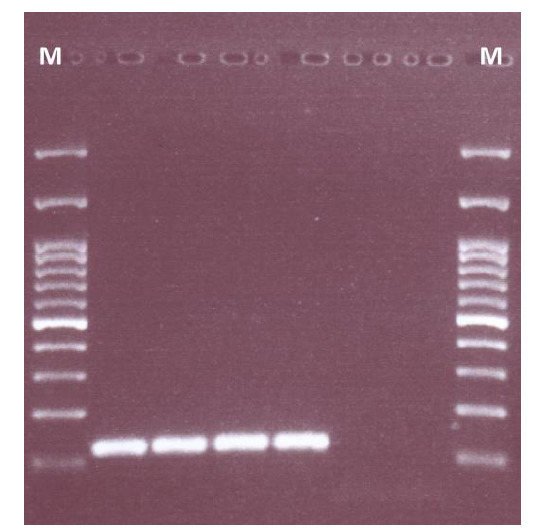
Agarose gel electrophoresis of *Candida auris* and *C. haemulonii* polymerase chain reaction products; M) 100-bp DNA size marker, lanes 1 and 2) *C. haemulonii* CBS 7801, lane 3) *C. auris* CBS 14916, lane 4) *C. auris* CBS 10913, and lanes 5 and 6) negative controls

Primers Cau-qRT-F and Cau-qRT-R successfully amplified the short ITS2 region of *C. auris* and *C. haemulonii* in conventional PCR reactions. The electrophoresis of the conventional PCR products revealed bands of 123 and 117 bp for *C. auris* and *C. haemulonii*, respectively (Figure 1). The amplification results showed 100% specificity for both *C. auris* and *C. haemulonii*, and no PCR products were detected for other fungal or bacterial strains or human DNAs. A temperature of 63°C was determined to be the best annealing temperature.

**Specificity and sensitivity of real-time polymerase chain reaction**

**Figure 2:**
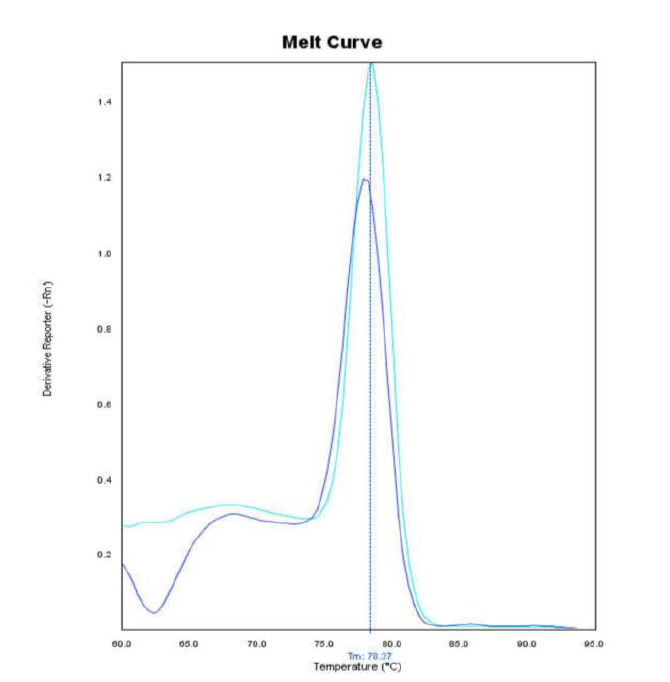
Melt curves of *Candida auris* and *C. haemulonii* real-time polymerase chain reaction products

Both *C. auris* and *C. haemulonii* DNAs produced valid amplification plots in SYBR Green-based real- time PCR reactions. The melting curve analysis of SYBR Green real-time PCR assays facilitated the accurate identification of and distinction between *C. auris* and *C. haemulonii*, with the melting temperatures (Tm) of 78.37°C and 77.99°C, respectively (Figure 2). Developed qPCR was able to selectively identify only C. auris DNA and produced reliable amplification plots. The DNA of *C. haemulonii* and all other fungal or bacterial strains, human DNAs, or NTCs and none of the clinical isolates produced amplification results.

**Figure 3:**
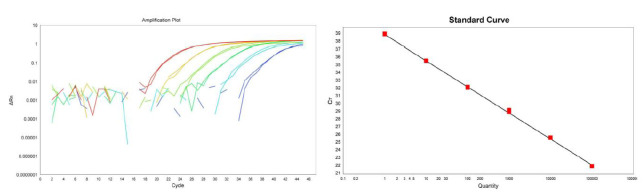
Left) linearity of hydrolysis probe-based quantitative polymerase chain reaction (qPCR) assays (10-fold serial dilutions of *C. auris* DNA were amplified.) and right) qPCR standard cure plotted with 10-fold serial dilutions of *C. auris* DNA

The standard curves in qPCR experiments revealed a slope of -3.428±-0.064 and an R2 of 0.994±0.004 with the efficiencies of 95.844±2.456 (Figure 3). The LOD was determined as 1 CFU of *C. auris* per 1 mL of sample, considering the coefficient of dilution and the DNA template volume (2 µL) used in LOD determination experiments.

## Discussion

*Candida auris* is one of the most opportunistic species of *Candida* that causes candidemia and invasive infections accounting for high morbidity and mortality rates [ 31 ]. The worldwide reports regarding the growth of *C. auris* infections now constitute a global health concern, and the US Centers for Disease Control and Prevention published a clinical alert about *C. auris* in June 2016 (https://www.cdc.gov/fungal/ diseases/candidiasis/candida-auris-alert.html).

Although *C. auris* is not very common among non- albicans *Candida* species, its increased incidence is very significant due to its multidrug-resistant properties [ 32 ]. The identification of the yeast with standard laboratory methods is difficult and may result in species misidentification without access to specific technologies. Moreover, the possible human or environmental reservoirs remain unknown at present. The global emergence of *C. auris* calls attention to the importance of using rapid, appropriate screening methods to detect and identify *C. auris* in infected patients [ 9 , 33 ].

Although sequencing or MALDI-TOF MS are promising tools for yeast identification, they require more expensive equipment than needed for real-time PCR. However, such kinds of technologies are not available in all developing countries. On the other hand, DNA amplification techniques, particularly PCR-based methods, have been increasingly used with success in recent decades for species identification. Currently, many reference and routine laboratories worldwide are equipped with thermal cyclers and real- time PCR machines. This makes it logical to focus on developing methods with optimum feasibility in as many of these laboratories as possible. In this study, conventional, SYBR Green-based, and quantitative TaqMan-based real-time PCR assays were tested in an effort to overcome the problem of identifying *C. auris* in clinical laboratories located in resource-limited regions.

This newly developed qPCR assay produced results within 3 h. Assay time for sample processing and DNA extraction was shorter than those of not only conventional methods but also other reported real-time PCR assays [ 26 , 27 ]. The conventional PCR assay clearly demonstrated that Cau-qRT-F and Cau-qRT-R primers produced specific bands for C. auris and

*C. haemulonii*. However, although this standard conventional PCR could help distinguish *C. auris* and *C. haemulonii* from other yeasts, it was unable to differentiate between these two species. Although routine laboratories can use these primers to identify the yeast in conventional PCR reactions, the full procedure, including DNA extraction, PCR, and electrophoresis, is time-consuming. Moreover, *C. auris* and *C. haemulonii* produced the amplicons of nearly the same size (123 and 117 bp, respectively), which cannot be differentiated with standard gel electrophoresis. Accordingly, we recommend using probe-based or SYBR Green-based real-time PCR to identify and distinguish these species.

Despite the fact that SYBR Green real-time PCR can detect both C. auris and C. haemulonii DNA, a melting curve analysis step is needed for differentiation, which is more time-consuming in comparison to the probe-based real-time PCR method. A further potential limitation is that the specific Tm for the PCR products of these species are very similar (78.37°C and 77.99°C, respectively), and this small temperature difference (0.38°C±0.05) may make interpretation challenging. Similar melting temperatures for the PCR products of *C. auris* and *C. haemulonii* were seen in a previously reported SYBR Green real-time PCR method introduced by Kordalewska et al. [ 26 ].

Consistent with our SYBR Green real-time PCR experiments, the difference between their reported Tm for the two PCR products (85.6°C±0.15 and 84.8°C±0.2 for *C. auris* and *C. haemulonii*, respectively) appears to be insufficient (∆Tm 0.8±0.35°C) to differentiate between these two related yeasts. Moreover, small Tm differences may be confounded with other variables during the different runs of real-time PCR assays.

Our hydrolysis probe-based qPCR assay showed high sensitivity and specificity for detecting and quantifying *C. auris*. The lower detection limit of the assay was 1 CFU of *C. auris* per mL, which is sensitive enough for use in both clinical management of patients and other healthcare-related tasks, such as hospital environmental monitoring. The present results suggested the probe-based real-time PCR assay as a reliable detection method, with a detection sensitivity of 1 CFU of *C. auris* per mL and a specificity of 100%. The limitations of this study were the lack of access to a suitable yeast bank in the country, delay in achieving the required standard species, and lack of enough clinical samples positive for *C. auris*. Moreover, the inability to directly determine fungal load from clinical sample was another limitation of using this real-time PCR assays method.

## Conclusion

*Candida auris* is a threatening organism for humans, and its laboratory diagnosis depends on the use of rapid and accurate methods. The method introduced in this study can improve the detection, identification, and quantification of *C. auris*.
